# Successful laparoscopic resection of fallopian tube abscess caused by Escherichia coli in a 12-year-old adolescent virgin:a case report and review of the literature

**DOI:** 10.1186/s12887-023-04098-8

**Published:** 2023-06-06

**Authors:** Xi-Feng He, Xiu-Ping Du, Cui-Feng Qiao

**Affiliations:** Master’s Degree, Department of Gynaecology And Obstetrics, Children’s Hospital of ShanXi, Women Health Center of ShanXi, TaiYuan, China

**Keywords:** Fallopian tube abscess, Adolescent virgin, Escherichia coli

## Abstract

**Background:**

Upstream infection with vaginal flora can develop into tubal endothelial damage and tubal edema, which can lead to tubal obstruction and fallopian tube abscess if left untreated. Fallopian tube abscess in adolescent virgins is very rare, it may lead to long-term or even lifelong complications once it occurred.

**Case presentation:**

A 12-year-old adolescent virgin with no history of sexual intercourse and previous physical fitness who presented with lower abdominal pain with nausea and vomiting for 22 h, body temperature up to 39.2 °C. Laparoscopic surgery revealed an abscess in the left fallopian tube, the left fallopian tube was surgically removed, successfully treated, and the pus was cultured for escherichia coli.

**Conclusion:**

It is important to consider possibility of tubal infection in young.

## Introduction

Fallopian tube abscess is a serious consequence of pelvic inflammatory disease, often due to upstream infection with vaginal flora cause tubal infection which can lead to tubal obstruction and fallopian tube abscess if left untreated [1]. Fallopian tube abscess is commonly seen in sexually active women of childbearing age [2].

Adolescence is the transitional period of development from the childhood stage to the adult stage, usually between the ages of 10 and 18 for girls, and the occurrence of pelvic inflammatory disease and fallopian tube abscess is very rare during this period if there is no history of sexual intercourse and is less frequently reported in the literature. Once they occur they may lead to long-term or even lifelong complications such as pelvic adhesions, chronic pelvic pain, infertility and ectopic pregnancy [3]. It is reported that the infectious source is typically polymicrobial and several reports have identified Escherichia coli(E.coli), *Neisseria gonorrhea*, and *Chlamydia trachomatis* and a variety of obligate anaerobic bacteria as commonly associated microorganisms. [4].

## Case description

**Fig. 1:**
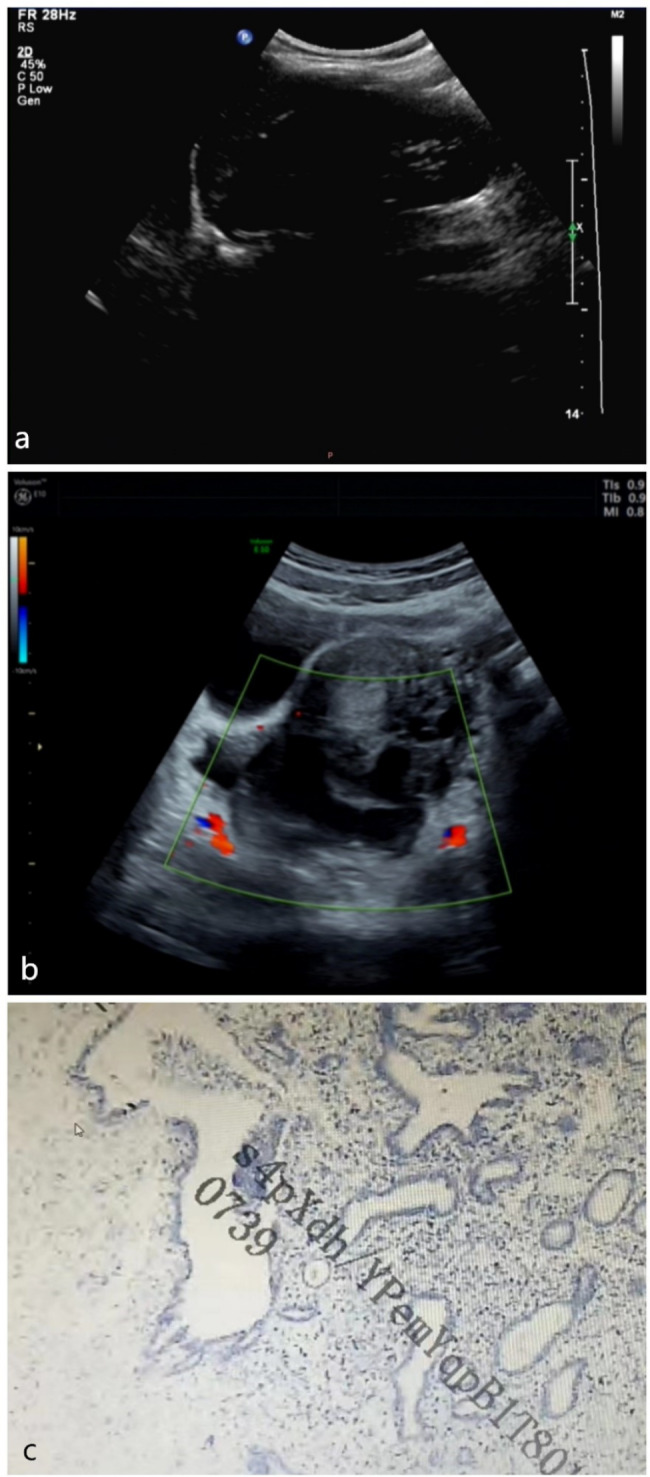
**a**: The first transabdominal pelvic ultrasound image. **b**: The second transabdominal pelvic ultrasound image. **c**: Histological pathological findings

A 12-year-old adolescent virgin with no sexual intercourse had lower abdominal pain with nausea and vomiting for 22 h, with persistent pain. Vomit is digestive fluid and food residue, and the lower abdominal pain was not significantly relieved after vomiting, body temperature is normal. Visit pediatric department, the transabdominal pelvic ultrasound was performed :A 9.7 × 3.8 cm cystic anechoic area with multiple band-like separations and a grid-like distribution was seen in the posterior left side of the uterus (Fig. 1-a). There was no history of abdominal pain, fever, night sweats, trauma, surgery or urinary tract infection, no increased vaginal discharge. Laboratory data showed a white blood cell (WBC) count of 13.98 × 10^9^/L, neutrophil percentage of 87.4%, and C-reactive protein (CRP) of 28.73 mg/L, all were above normal. The body temperature was 36.7 °C, pulse 128/min, respiration 25/min, blood pressure125/79mmHg, abdomen was flat, drum sound on percussion of the abdomen, lower abdomen was tense, there was tenderness on palpation, rebound tenderness was.

Suspicious and no obvious mass was palpable throughout the abdomen. The diagnosis of admission was unclear.

Body temperature gradually increased to 38 °C during the patient’s transfer to the gynecology department and the repeated transabdominal pelvic ultrasound was performed: The tortuous tubular anechoic area, about 7.2 × 4.8 cm in extent, with a thick and rough wall and poor internal translucency was seen in the posterior left side of the uterus (Fig. 1-b). After patient referred to gynecology, the body temperature rises to 39.2℃. The anal examination: a cystic mass could be palpated in the posterior part of the uterus, the size was9 × 8 cm, the pressure pain was positive. The WBC count was 12.89 × 10^9^/L, neutrophil percentage was 85.3%, CRP was 124.48 mg/L, procalcitonin (PCT) was 4.88 ng/ml, erythrocyte sedimentation rate (ESR) was 60 mm/h, all were above normal. In combination with gynecologic ultrasound, pelvic abscess and ovarian cyst torsion combined with infection were not excluded. She was treated with intravenous cefoperazone sodium and metronidazole.

**Fig. 2:**
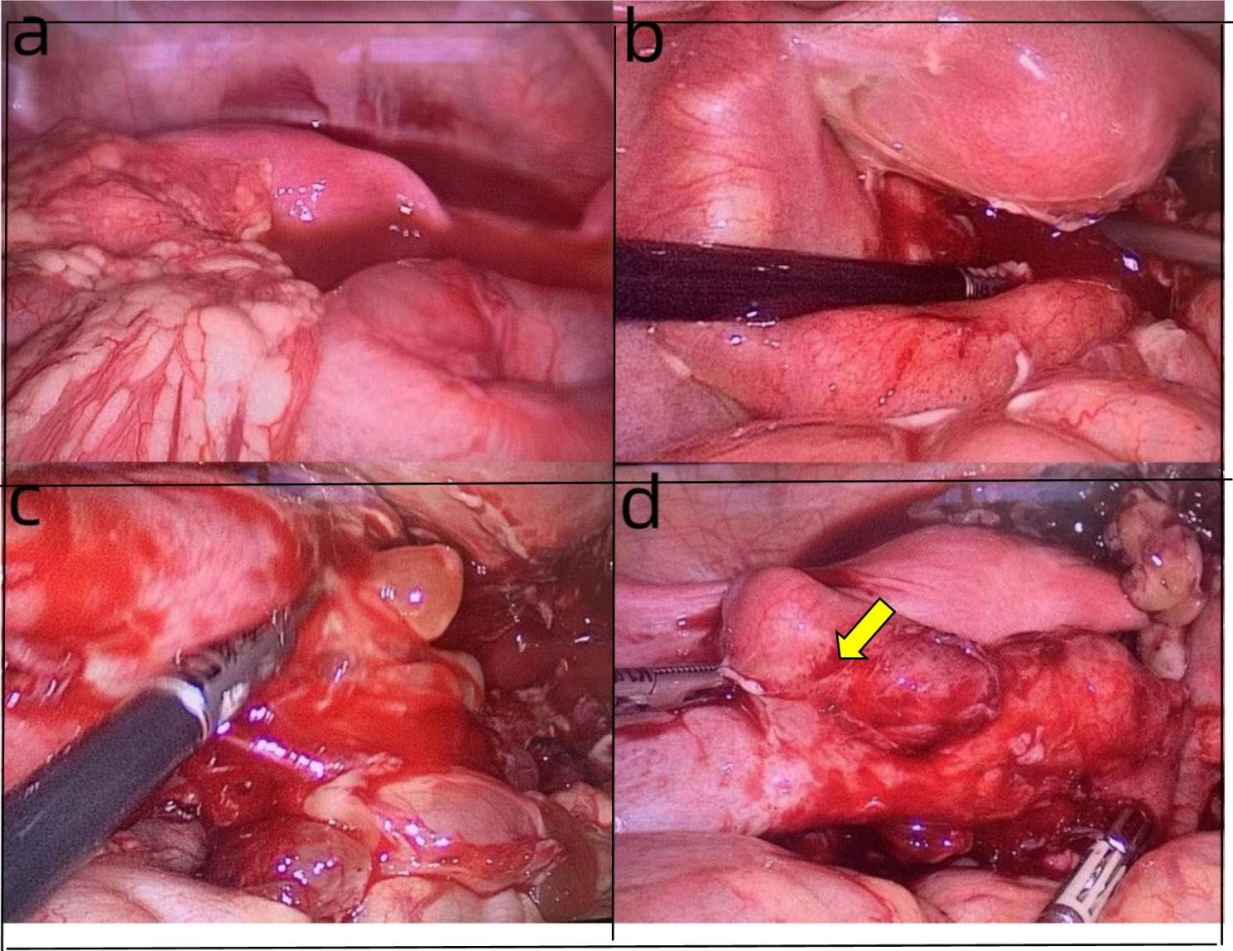
Intraoperative laparoscopic images, removed left fallopian tube indicated by arrow

General surgery consultation was requested and pelvic enhancement computed tomography (CT) was recommended. Since pelvic enhancement CT could not be performed during nighttime, the patient was considered to have significant acute abdomen and fever, and laparoscopic exploration was performed urgently. Laparoscopic examination showed that the anterior uterus and intestinal space were covered with pus and blood fluid, amounting to about 100 ml, and the intestines and large omentum were densely adherent to the posterior wall of the uterus and bilateral adnexa, and the Douglas fossa was completely closed(Fig. 2-a), and pus was extracted for bacterial culture. After separation of adhesions see the right fallopian tube and ovary were normal, while the left fallopian tube was obviously thickened, about 2.5 cm in diameter, hard, brittle and poorly mobile, with a blind end at the umbrella end and dense adhesions with the left ovary and posterior wall of the uterus(Fig. 2-b). A purulent mass of about 7 × 6 cm in size was seen in the left adnexal area of Douglas’ fossa, the bad and brittle tissue of Douglas’ fossa was removed (Fig. 2-c), the left ovary was exposed to be 3 × 2 cm in size, with pus moss visible on the surface. Considering a left fallopian tube abscess, the left fallopian tube was removed and pelvic irrigation was performed (Fig. 2-d).

**Chart 1:**
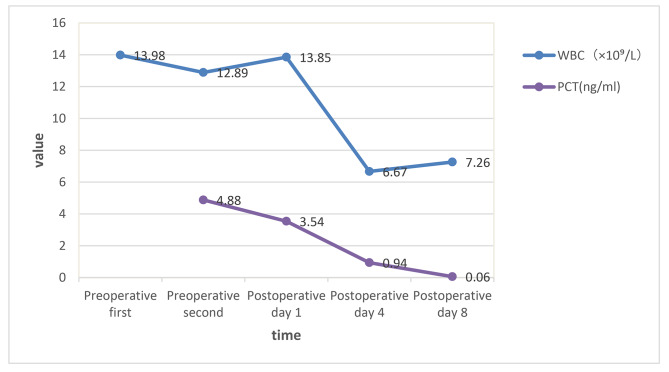
Pre-operative and post-operative WBC and PCT change line chart

**Chart 2:**
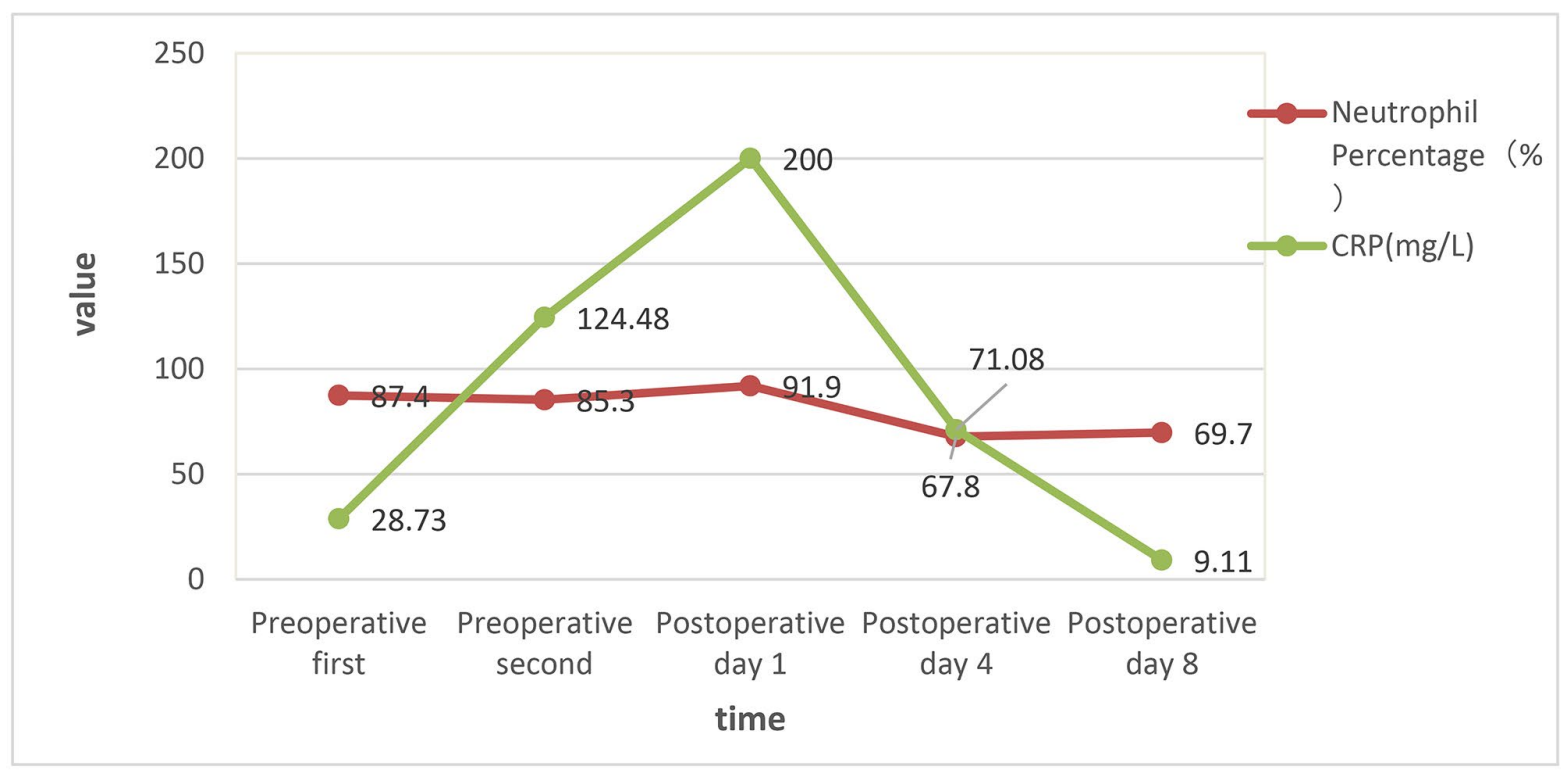
Pre-operative and post-operative Neutrophil Percentage and CRP change line chart

After the operation, the body temperature dropped rapidly to normal, and the patient was treated with two intravenous antibiotics: cefoperazone sodium for 12 days at a dose of 1 g/dose every 8 h, and metronidazole for 5 days at a dose of 100 ml/dose every 8 h. The postoperative course was stable, and WBC count, neutrophil percentage, CRP and PCT all returned to within the normal range(Table 1, Chart 1, 2), and she was discharged 12 days after surgery. Bacterial culture of pus: E. coli. Histological pathological findings: acute purulent tubal inflammation with perforation of the left fallopian tube (Fig. 1c).

**Table 1:** Pre-operative and post-operative laboratory data value

	Preoperative first	Preoperative second	Postoperative day 1	Postoperative day 4	Postoperative day 8
WBC(×10^9^/L)	13.98	12.89	13.85	6.67	7.26
Neutrophil Percentage(%)	87.4	85.3	91.9	67.8	69.7
CRP(mg/L)	28.73	124.48	200	71.08	9.11
PCT(ng/ml)		4.88	3.54	0.94	0.06

The patient was 150 cm tall and weighed 44.5 kg. The first menstruation occurred at the age of 12 years, 4 months before admission. The menstrual cycle was 20 days, the period was 7 days, the volume was moderate, and the dysmenorrhea was negative.

## Discussion and conclusion

Fallopian tube abscesses in sexually inactive adolescent are very rare, this is the second case of fallopian tube abscess in adolescent virgins caused by E. coli in which no causative high-risk factors could be found. Fallopian tube abscesses can present clinically with fever, chills, nausea, vomiting, lower abdominal pain, abnormal vaginal bleeding, vaginal discharge [3, 5], and in severe cases can lead to diffuse peritonitis, sepsis, and infectious shock. It can also lead to distant complications, which can affect the quality of life in the long term [3]. Therefore, once the above symptoms appear, early and rapid diagnosis and treatment is the key to reduce lifelong complications.

It is reported that CT has a higher sensitivity for the diagnosis of tubo-ovarian abscesses and to differentiate the disease from similar gastrointestinal pathology [6]. Ultrasonography is the first-line imaging modality in the evaluation of acute gynecologic disease, however, CT can narrow the differential diagnosis when the diagnosis cannot be established [7]. Due to limitations, pelvic CT was not performed on admission in this case. Previous studies have shown that abscesses larger than 6.5 cm and fever are independent predictors of the need for surgical treatment in patients with fallopian tube abscesses [8]. In this patient, on admission, ultrasound was performed: A 9.7 × 3.8 cm cystic anechoic area was seen on the posterior left side of the uterus. The repeated gynecologic ultrasound was performed: The tortuous tubular anechoic area, about 7.2 × 4.8 cm in extent was seen in the posterior left side of the uterus. The anal examination: a cystic mass could be palpated in the posterior part of the uterus, the size was 9 × 8 cm, the pressure pain was positive. The pelvic abscesses were not excluded and all were suggestive of greater than 6.5 cm. Other studies have shown the reliability of ESR in predicting infection in the diagnosis of fallopian tube abscesses [9]. The recommended threshold value of ESR is 61.0 mm/h [10]. CRP level is the most reliable parameter for the diagnosis of pelvic infections and it represents the best criterion to assess the effectiveness of treatment for patients with pelvic infections [11]. CRP is an acute phase reactant produced by the liver in response to infection and/or inflammation. PCT is the pre-hormone of calcitonin and it is secreted by the C cells of the thyroid gland PCT is a useful marker in the diagnosis of systemic infection and sepsis. A cutoff level of 0.330 ng/ml for PCT revealed 62% sensitivity and 75% specificity in predicting tubo-ovarian abscesses [12]. In our study, the patient was admitted with high WBC count and neutrophil percentage, CRP 124.48 mg/L, PCT 4.88ng/ml, ESR 60 mm/h, all these infection indicators suggest serious pelvic infection. In conclusion, the patient was admitted with a temperature of 39.2 °C and an acute abdomen, with infection indicators suggesting severe infection, and combined with ultrasound suggesting a possible pelvic abscess larger than 6.5 cm, with indications for surgery, and laparoscopic surgery was quickly selected along with antibiotic treatment, and intraoperatively the left fallopian tube abscess was seen, and the left fallopian tube was removed, the pelvic pus was removed, and the pelvic cavity was flushed. After the operation, the patient’s temperature dropped rapidly to normal. The rapid diagnosis and surgical treatment were timely and effective.

**Table 2:** Review of the pelvic abscess cases reported to date in virginal adolescent girls. (Revised from Maraqa et al. [14])

No.	Authors	Year of case publication	Age (years)	Symptoms	Postoperative diagnosis	Possible causal factors	Species
1	Sirotnak et al. [14]	1996	12	Right lower abdominal pain, emesis	FTA	None	S. pneumoniae
2	Sirotnak et al. [14]	1996	12	Bilateral lower abdominal pain, difficulty breathing, menstruation	FTA	None	S. pneumoniae
3	Pomeranz et al. [15]	1997	15	Abdominal pain	FTA	Relapsing Henoch-Schönlein purpura	Morganella morganii
4	Algren and Strickland [14]	2005	14	Lower abdominal pain, fever, dysuria, night sweats, nausea, vomiting, diarrhoea	FTA	None	Streptococcus group F, Fusobacterium nucleatum
5	Lerand and Jay [14]	2007	12	General abdominal pain, fever	FTA	Obesity, type II diabetes, UTIs, constipation	E. coli
6	Lerand and Jay [14]	2007	16	Right lower abdominal pain, suprapubic pain, fever, chills, anorexia	FTA	Candida vaginitis, Crohn’s disease	E. coli
7	van der Putten et al. [14]	2008	11	Abdominal pain, nausea, fever	FTA	None	S. pneumoniae
8	Singh-Ranger et al. [14]	2008	17	Lower abdominal pain, back pain, appetite loss, rigours	FTA	Appendectomy	E. coli
9	Hornemann et al. [14]	2009	13	Right lower abdominal pain, fever	FTA	None	E. coli
10	Desai and Ward [14]	2011	12	Bilateral and suprapubic abdominal pain, fever, emesis, vaginal discharge	FTA	Hirschsprung’s disease (colectomy), appendectomy, tonsillectomy	None
11	Moralioğlu et al. [14]	2013	14	Abdominal pain, vomiting, fever	FTA	Anal atresia with rectovestibular fistula, sigmoidectomy, uterus bicornis unicollis, septate vagina	E. coli
12	Kielly and Jamieson [14]	2014	11	Right lower abdominal pain radiating throughout abdomen, nausea, emesis	FTA	Constipation, encopresis	Unknown
13	Schmieg et al. [14]	2014	12	Lower abdominal pain, nausea, vomiting.	FTA	Appendectomy	E. coli
14	Maraqa et al. [14]	2017	12	Lower abdominal pain, fever, nausea	FTA	Obesity, UTIs, IBS, dilated vagina (Mullerian duct anomaly)	Streptococcus anginosus, Peptostreptococcus anaerobius, Prevotella bivia

UTI: urinary tract infection, FTA:fallopian tube abscess, IBS: irritable bowel syndrome

Some studies have shown that the genital tract of the prepubescent child is different from that of a woman of reproductive age. Unlike the normal acidic pH of the adult female vagina, the pH of the premenarchal vagina is neutral. [13]This creates an environment that may facilitate overgrowth of the normal vaginal flora (E.coli, Gardnerella vaginalis, Staphylococci, Streptococci, and so on). In addition, it lacks the vaginal antibodies that may appear later in life [13]. These two factors may increase the susceptibility for vaginal infection in children. In this review of the literature, the most common causative organism of fallopian tube abscesses in adolescent virgins was E.coli, followed by S.pneumoniae, and occasionally Morganella morganii, Streptococcus group F,*Fusobacterium nucleatum*, Streptococcus anginosus, Peptostreptococcus anaerobius, Prevotella bivia. (Table 2) [14, 15]. The causative agent of tubal abscess in this case was E. coli. Traditionally, broad-spectrum intravenous antibiotic infusion is the preferred treatment for pelvic abscess. In this case, broad-spectrum antibiotic cefoperazone sodium and metronidazole for anaerobic bacteria were chosen and the antibiotics were effective for their treatment. The blood picture, CRP and PCT decreased to normal.

Fallopian tube abscesses in adolescent virgins are easily misdiagnosed because of the lack of many factors of ascending infection, although they exhibit clinical symptoms. In this study, literature review (up to 2022) of fallopian tube abscesses in adolescent virgins included 14 cases (Table 2) [14, 15]. This suggests that fallopian tube abscesses rarely occur in adolescent virgins. The cause of fallopian tube abscesses in this study is unknown. Common high risk factors in the literature review are: urinary tract infection ascending infection, congenital genitourinary anomalies, crohn’s disease, tonsillectomy, adjacent organs such as appendectomy, colectomy, constipation, encopresis, irritable bowel syndrome, obesity, diabetes mellitus, and so on.

## Conclusion

In conclusion, It is important to consider possibility of tubal infection in young women with acute abdomen and fever, even if they have never had sexual relations, although there are no high-risk factors. E. coli may be the causative agent, and early and timely diagnosis and treatment are essential to prevent future sequelae.

## Data Availability

The datasets used and/or analysed during the current study available from the corresponding author on reasonable request.

## Abbreviations

E.coli: Escherichia coli
WBC: white blood cell
CRP: C-reactive protein
PCT: procalcitonin
ESR: erythrocyte sedimentation rate
CT: computed tomography
UTI: urinary tract infection
FTA: fallopian tube abscess
IBS: irritable bowel syndrome
